# Exploring Occupational Therapists’ Sleep Intervention Approaches and Perspectives for Children With Autism Spectrum Disorder: A Malaysian Qualitative Study

**DOI:** 10.1155/oti/5593400

**Published:** 2026-04-27

**Authors:** Muhammad Radhi Rahimi Abu Bakar, Akehsan Dahlan, Zul-′ Izzat Ikhwan Zaini, Hanif Farhan Mohd Rasdi

**Affiliations:** ^1^ Occupational Therapy Program, Faculty of Health Sciences, Universiti Teknologi MARA Cawangan Pulau Pinang, Bertam Campus, Kepala Batas, Pulau Pinang, Malaysia; ^2^ Centre for Occupational Therapy, Faculty of Health Sciences, Universiti Teknologi MARA Cawangan Selangor, Puncak Alam Campus, Bandar Puncak Alam, Selangor, Malaysia; ^3^ Department of Basic Science, Faculty of Health Sciences, Universiti Teknologi MARA Cawangan Pulau Pinang Bertam Campus, Kepala Batas, Pulau Pinang, Malaysia; ^4^ Program of Occupational Therapy, Faculty of Health Sciences, Universiti Kebangsaan Malaysia, Kuala Lumpur, Malaysia, ukm.my

**Keywords:** autism spectrum disorder, children, Malaysia, pediatric occupational therapist, sleep interventions

## Abstract

**Introduction:**

Children with autism spectrum disorder (ASD) frequently experience significant sleep disturbances, adversely affecting their health and family well‐being. Despite the high prevalence, limited research exists on sleep interventions employed by pediatric occupational therapists (OTs) in Malaysia. This study explores the types of sleep interventions used and the perspectives of pediatric OTs in managing sleep disturbances among children with ASD.

**Methods:**

An exploratory qualitative design was adopted. Data were collected through two focus group discussions (FGDs) with 10 pediatric OTs from private rehabilitation centers in northern Malaysia. Thematic analysis followed Braun and Clarke’s six‐step process, with researcher reflexivity and collaborative coding employed to ensure rigor.

**Results:**

Participants identified a multimodal approach including behavioral strategies (e.g., visual schedules), sensory‐based techniques (e.g., deep pressure), physical activity, and environmental modifications. A structured clinical decision pathway emerged, prioritizing sensory regulation as a physiological prerequisite for behavioral success. Parental involvement was highlighted as critical, though moderated by socioeconomic and cultural factors such as cosleeping. Notably, therapists perceived improvements in children’s sleep quality and reductions in parental stress.

**Conclusion:**

This study underscores the value of a culturally responsive and modular approach to sleep interventions within the private pediatric OT landscape of northern Malaysia. The findings provide a foundational framework for future quantitative research to establish objective effectiveness. The results advocate for enhanced awareness, specialized training, and framework‐aligned assessment tools to support families and therapists.

## 1. Introduction

Children with autism spectrum disorder (ASD) often face a range of challenges that can significantly affect their daily lives, including difficulties in communication, social interactions, and behavior regulation [1]. One of the most prominent and disruptive challenges experienced by children with ASD is sleep disturbances [2, 3], which affect not only the child’s health but also their family’s well‐being [4, 5]. Research has shown that sleep disturbances are more prevalent in children with ASD than in the general population, with estimates suggesting that between 40% and 80% of children with ASD experience significant sleep disturbances [2, 6–8]. These issues, which often include difficulties falling asleep, staying asleep, or waking up too early [9–11], can exacerbate other behavioral symptoms, leading to increased irritability, hyperactivity, and reduced ability to focus, thus affecting a child’s functioning at home and in school [8, 12]. Furthermore, these sleep disturbances not only impact the child but also place a significant emotional and physical strain on families. Caregivers of children with ASD often report heightened levels of stress, poor sleep quality themselves, and challenges in managing daily routines due to the lack of consistent sleep from their children [3, 8]. As a result, addressing sleep issues in children with ASD is essential for both the child’s development and the overall well‐being of the family.

Comprehensive sleep assessments and targeted interventions are often lacking for children, particularly those with ASD [13, 14]. These difficulties are usually compounded by communication challenges, which frequently result in their sleep disturbances being misinterpreted, misdiagnosed, or left untreated due to difficulties in expressing sleep‐related discomfort [6, 15]. Given these limitations, pediatric occupational therapists (OTs) are uniquely positioned to conduct early assessments and provide appropriate interventions that align with the child’s developmental and sensory needs. Despite the high prevalence of sleep disturbances in children with ASD, the specific approaches used by pediatric OTs remain underdocumented, particularly within Malaysia [9, 16–18].

The Occupational Therapy Practice Framework (OTPF, fourth edition) provides a professional guideline for understanding how various factors influence a child’s ability to engage in daily activities, including sleep [19]. In the case of children with ASD, the *OTPF-4* emphasizes the interaction between the child’s body functions, the environment, and the activity demands, which together impact their sleep patterns. Specifically, performance skills such as sensory processing and self‐regulation play a critical role in sleep. Children with ASD may exhibit difficulties in sensory processing, such as heightened sensitivity to environmental stimuli (e.g., light, noise, or textures), or sensory‐seeking behaviors, which can affect their ability to transition into sleep effectively [16, 20, 21]. These sensory challenges may disrupt sleep routines and contribute to insomnia or frequent nighttime waking. The *OTPF-4* also highlights the importance of addressing environmental factors and personal factors that influence a child’s ability to engage in restorative sleep. For children with ASD, environmental modifications such as adjusting lighting, minimizing noise, and organizing room layout can play a vital role in creating a sleep‐conducive setting that reduces overstimulation and anxiety [8, 21].

Pediatric OTs have long been involved in addressing various challenges faced by children with ASD, including issues related to sleep [20]. OTs utilize a range of therapeutic interventions aimed at promoting self‐regulation, enhancing daily routines, and modifying the child’s environment to improve sleep quality. Commonly employed interventions include behavioral strategies, sensory‐based techniques, physical activity, and environmental adjustments [20, 22, 23]. However, while numerous studies have examined the role of OTs in managing sleep disturbances in children with ASD, there remains limited research on the specific strategies and interventions used by pediatric OTs in Malaysia. While numerous sleep interventions have been developed and tested in Western countries, these strategies often assume cultural norms such as independent sleeping arrangements and consistent bedtime routines. This gap is particularly important given the cultural, socioeconomic, and healthcare context in Malaysia, which may influence both the nature of sleep disturbances and the types of interventions that are most effective in addressing them. Furthermore, it is important to note that sleep practices such as cosleeping are more common in Eastern cultures, including Malaysia, compared to Western countries, where independent sleeping is often emphasized. This cultural difference has implications for occupational therapy practices, as intervention strategies developed in Western contexts may not fully align with Malaysian family norms. Adapting sleep interventions to accommodate cosleeping arrangements while promoting healthy sleep routines could improve their acceptability and effectiveness in the local context [9, 24]. As such, understanding how pediatric OTs in Malaysia address sleep disturbances in children with ASD is essential for developing effective intervention strategies. While this study provides a preliminary exploration of sleep interventions in Malaysia, it specifically focuses on the practices within private pediatric occupational therapy centers in the northern region. This focus allows for a detailed look at resource‐rich environments, which may differ significantly from the public healthcare system or community‐based rehabilitation centers (CBRCs) across the country. Therefore, this research is aimed at exploring the types of sleep interventions used by pediatric OTs in Malaysia and understanding their perspectives in addressing sleep difficulties among children with ASD.

## 2. Methods

This study employed an exploratory qualitative design to examine the sleep intervention practices of pediatric OTs working with children diagnosed with ASD in Malaysia.

### 2.1. Researcher Characteristics and Reflexivity

The primary researcher and moderator is a lecturer and a male doctoral candidate in occupational therapy with more than 10 years of clinical experience in pediatric rehabilitation. Throughout the study, the researcher maintained a reflexive journal to identify and mitigate personal biases, ensuring that the facilitation of focus groups remained neutral. The researchers’ prior clinical familiarity with the centers assisted in establishing rapport with participants, while the collaborative coding process among the multi‐investigator team—including experts in qualitative health research—ensured that the findings were grounded strictly in the participants’ data rather than the researchers’ preconceptions.

### 2.2. Participants

This study explored pediatric OT sleep interventions for children with ASD in Malaysia. Given the limited research in this area, an exploratory qualitative design was employed as the theoretical framework for this study to gain a deeper understanding of the types of interventions used and the challenges faced by pediatric OTs. Ten participants, aged between 25 and 36 years (M = 30.4; SD = 4.27), with four males and six females, participated in the study. The inclusion criteria for participants were (1) OTs aged 20 years and above, (2) holding at least a diploma and up to a doctoral degree, and (3) having a minimum of 12 months’ experience in working with children with ASD. Educational level was reported as follows: Six participants were bachelor’s degree holders, while three had a diploma, and one participant had a master’s degree. The years of experience in the pediatric area were as follows: 7–10 years (*n* = 2), 3–6 years (*n* = 6), and 1–2 years (*n* = 2). The sociodemographic characteristics of the participants are illustrated in Table 1.

**Table 1:** Sociodemographic characteristics of the participants.

Participant ID	Gender	Age	Educational level	Years of experience working with children with ASD	Specialization	Location
Participant 1	Female	34	Bachelor’s degree	10 years	Pediatric	Penang
Participant 2	Male	31	Bachelor’s degree	2 years	Pediatric	Penang
Participant 3	Male	36	Master’s degree	7 years	Pediatric	Penang
Participant 4	Male	25	Diploma	4 years	Pediatric	Perlis
Participant 5	Female	27	Bachelor’s degree	5 years	Pediatric	Perlis
Participant 6	Female	33	Diploma	2 years	Pediatric	Perlis
Participant 7	Female	36	Bachelor’s degree	6 years	Pediatric	Penang
Participant 8	Female	27	Bachelor’s degree	4 years	Pediatric	Perlis
Participant 9	Female	30	Bachelor’s degree	4 years	Pediatric	Penang
Participant 10	Male	25	Diploma	3 years	Pediatric	Perlis

### 2.3. Recruitment

To reach the intended population, potential participants were recruited through purposive and convenience sampling targeting pediatric OTs. The participants were recruited from two pediatric rehabilitation centers in the northern region of Malaysia, one in Penang and one in Perlis. Both of these centers are privately funded and serve early intervention programs (EIPs) and rehabilitation programs for special needs, especially children with ASD. The occupational therapy services at these centers include group therapy, sensory integration, behavioral training, EIP, and parent education. Typical sessions ranged from 60 min to 3 h, with frequencies of once to three times per week, depending on each child’s needs, progress, or parents’ request. The recruitment from these two privately funded centers reflects a specific segment of Malaysian pediatric practice where therapists may have more flexibility in session length and frequency compared to the high‐volume public sector. Permission to recruit participants was granted by both centers prior to the recruitment process, and the study received approval was obtained by the Research Ethics Committee (REC), University Teknologi Mara (REC/02/2025) (ST/MR/24). Recruitment information was given to the center supervisor at these two centers for participant recruitment. Supervisors were instructed to distribute the information to OTs who had much experience with children with ASD. The recruitment information outlined the study details and eligibility criteria and provided the research team’s contact information for verifying study eligibility. To meet the objectives of the study, data collection was conducted in a focus group, with an anticipated length of between 45 and 60 min.

### 2.4. OT Focus Group

Data were collected through two focus groups, each with five participants, using a semistructured guide. All data were collected through two face‐to‐face focus group discussions (FGDs), each consisting of five OTs. A semistructured interview guide was used to moderate the group discussions, not individual interviews. The choice of FGDs was intentional, aiming to explore the collective clinical reasoning and professional consensus among Malaysian pediatric OTs, rather than individual idiosyncratic practices. This format allowed for a dynamic exchange where participants could build upon or challenge each other’s perspectives on sleep intervention strategies. Focus groups are a valuable data collection method for understanding and exploring specific topics, providing in‐depth conversations and interactions among individuals [25]. The sample size of 10 participants, divided into FGDs, was deemed sufficient to capture the breadth of clinical practices within the specialized private sector of northern Malaysia. Data saturation was determined when no new strategies, barriers, or perspectives emerged during the second focus group. Iterative coding of the transcripts revealed thematic redundancy, suggesting that the data sufficiently addressed the research objectives within the defined clinical scope. During this enrollment phase, participants’ eligibility for the study was verified. The participants were informed multiple times that their responses would be anonymized to maintain confidentiality. Written informed consent for audio recording and participation in FGDs was obtained before data collection. The structured questionnaire captures demographic and professional background information (e.g., age, gender, educational qualification, and years of experience in pediatric occupational therapy). This was used to describe the sample characteristics and contextualize their perspectives. Once the consent form and questionnaire were completed, focus groups were arranged according to the participants’ preferred date and time for both of these centers. The FGDs were conducted in person at the participating rehabilitation centers. A trained moderator (the first author) facilitated both sessions, while an assistant researcher managed audio recording and field notes. Discussions followed a semistructured FGD guide adapted from guidelines by [25], which recommend open‐ended prompts, facilitator neutrality, and group interaction management. The FGDs were recorded both audibly, using an audio recorder, to support data analysis. The data transcription was employed and reviewed for accuracy immediately after the FGDs. The focus groups were conducted in Malay using the semistructured interview guide. This semistructured interview guide was designed to facilitate the conversation and ensure that essential topics related to the participants’ experiences were thoroughly explored. These discussions provided deeper insights into their experiences when implementing sleep interventions for children with ASD. The data from these focus groups were analyzed using thematic analysis to identify key themes and subthemes related to the types of interventions, structure, and the perspectives of OTs. For publication, relevant quotations were translated into English by a bilingual researcher with expertise in qualitative health research. To ensure accuracy and preserve conceptual meaning, back‐translation was performed independently by a second bilingual expert, and discrepancies were resolved through discussion.

### 2.5. Data Analysis

Focus group transcripts were subjected to thematic analysis to explore in‐depth experiences and perceptions related to the implementation of sleep interventions. The audio recordings were transcribed verbatim and reviewed for accuracy. Thematic analysis was conducted following Braun and Clarke’s [26] six‐step process. Both key themes and subthemes were identified through iterative coding. Initial open codes were grouped into categories, which were refined into themes and subthemes aligned with the study objectives. Initially, the transcripts were read thoroughly to gain familiarity with the data. Then, open coding was applied to identify meaningful units reflecting barriers and facilitators described by participants. Codes were grouped into broader categories, which were subsequently refined into key themes and subthemes corresponding to the study objectives. To enhance rigor and trustworthiness, the coding process was conducted independently by three members of the research team, and discrepancies were resolved through discussion until consensus was reached. A coding framework was developed and used to recode the data to ensure consistency [27, 28]. Member checking and peer debriefing were employed by sharing summaries of key themes with participants to verify the accuracy and resonance of the findings, allowing them to confirm or clarify interpretations [29]. NVivo software (Version 14) was utilized to organize and manage qualitative data efficiently and for an audit trail. Triangulation was achieved through the integration of multiple coders and repeated review cycles.

## 3. Results

Thematic analysis of the data collected from two FGDs with participants regarding sleep intervention in children with ASD provides a detailed understanding of their experiences and perceptions, with durations of 48 and 61 min, respectively. Several key themes and subthemes emerged during the analysis, offering insight into the types of interventions and perspectives on implementing sleep interventions in this population. The key themes that emerged are illustrated in Table 2.

**Table 2:** Theme, subtheme, and codes.

Theme	Subtheme	Codes
Intervention implementation and strategy	Structure of the intervention	● Regularity
		● Frequency
		● Consistency
	Types of sleep intervention implemented	● Behavioral intervention
		● Sensory‐based intervention
		● Physical activity
		● Environmental strategies
Perspective on the intervention	Belief in the intervention	● Parent empowerment and consistency
		● Sensory regulation for sleep
		● Belief in improved sleep through activity
	Benefits of the intervention	● Improved sleep quality and routine
		● Increased functionality and academic performance
		● Reduction in parental stress and anxiety
	Value of the intervention	● Importance of routine and consistency
		● Guidelines and structured approaches
		● Targeted interventions for specific needs
Collaborative delivery of sleep intervention	Referral practices	●Specialized treatment and medical diagnosis

### 3.1. Theme 1: Intervention Implementation and Strategy

This theme explores how participants implement and strategize sleep interventions for children with ASD. It covers the structure of the interventions, the types of sleep interventions implemented, and the types of sleep interventions suggested by participants. These strategies are tailored to meet each child’s unique needs, often involving adjustments to routines, behavior, and environment. Additionally, participants suggest complementary therapies or medical referrals when necessary to enhance the effectiveness of interventions.

#### 3.1.1. Subtheme 1: Structure of the Intervention

This theme explores the regularity, frequency, and consistency of the interventions provided. The participants reported that the frequency and intensity of interventions were largely shaped by factors such as available time, external constraints (e.g., appointment schedules), and parental feedback. The findings revealed that interventions were tailored to meet individual needs and were often adjusted based on the severity and persistence of the child’s sleep disturbances.

##### 3.1.1.1. Code: Regularity of Intervention.

One participant emphasized the importance of maintaining regular intervention strategies, such as screen time limitations and environmental adjustments. These interventions were applied consistently, particularly in the period leading up to bedtime, in order to promote better sleep hygiene.

For example, we limit screen time to at least 2 hours before sleep, ensuring no screens are used at all. We also prepare the room by dimming the lights. (Participant 7).These interventions were implemented consistently as part of a daily routine, focusing on behavioral strategies like limiting screen time and creating a sleep‐friendly environment. Such adjustments are crucial for setting up the conditions that foster better sleep patterns.

##### 3.1.1.2. Code: Frequency of Intervention.

The frequency of intervention varied based on the setting and patient availability. For some participants, the intervention was delivered frequently, with some patients receiving daily sessions, while others had sessions once a week due to logistical constraints like appointment availability.

At our facility, we offer two types of sessions—group and individual. Individual sessions are offered more frequently, usually once a week, ranged from 60 minutes to 3 hours. (Participant 7)

At our center, we typically offer sessions daily or about three times a week. When patients come in frequently, we focus on educating them about sleep. (Participant 1)In institutional settings, participants could offer more frequent, individual interventions, while group interventions were scheduled weekly. However, the frequency of interventions was often contingent on the availability of patients and the structure of the treatment facility. For instance, sensory diets or behavioral adjustments were often reviewed after a 1–2‐week implementation period to determine if the routine had stabilized or required further modification. This suggests that the intensity of the intervention is defined more by its daily home consistency than by the frequency of clinic visits.

##### 3.1.1.3. Code: Consistency of Intervention.

The consistency of the interventions was largely driven by parental feedback and concerns about their children’s sleep issues. Participants acknowledged that parental involvement and reporting were key to maintaining continuous intervention. When parents raised concerns about their child’s sleep difficulties, participants provided follow‐up interventions to track progress and ensure the effectiveness of the sleep strategies.

How often do we conduct interventions? It depends on the frequency of issues raised by parents. If they report sleep problems or difficulties getting their child with autism to sleep, we will provide interventions. (Participant 3)

It’s the same approach of sensory diets or sleep hygiene; when there’s a complaint, we will follow up. We will ask, ′Did the child sleep better last night?′ After doing this for a week or two, we′re going to check if their sleep routine has improved or stayed the same. (Participant 2)These follow‐up interactions were vital for maintaining the consistency of interventions, ensuring that any required adjustments could be made and that parents remained involved in the process.

#### 3.1.2. Subtheme 2: Types of Sleep Interventions Implemented

OTs in Malaysia utilize various interventions to address sleep disturbances in children with ASD. These interventions include behavioral, sensory‐based, physical, and environmental strategies, which are tailored to meet the individual needs of the children. The following sections describe the types of sleep interventions implemented based on the data from the participants.

##### 3.1.2.1. Code: Behavioral Intervention.

Behavioral interventions focused on communication strategies and establishing presleep routines for children with ASD. Given the communication challenges of children with ASD, some participants utilized the Picture Exchange Communication System (PECS) or visual schedules to help children “see” the sequence of the bedtime routine. These tools helped children engage with sleep‐related activities and routines.

For children with autism, many of them have difficulty communicating verbally, so we use visual cues or PECS as an alternative method of communication. (Participant 3)Additionally, participants worked with children and parents to correct routines and suggested calming presleep activities when routines were disrupted.

We suggest activities before bedtime to help calm the child, especially when their routine changes and they have difficulty falling asleep. (Participant 2)The participants also emphasized educating parents about the negative consequences of poor sleep hygiene, particularly in children with ASD, and the impact on their child’s behavior and development.

We emphasize educating parents about the negative consequences of poor sleep hygiene and how it can affect their child′s behavior and development. (Participant 6)

##### 3.1.2.2. Code: Sensory‐Based Intervention.

Sensory‐based interventions were implemented to address sensory sensitivities that impacted sleep. These included sensory activities such as tactile input and proprioceptive exercises, which helped children regulate their sensory experiences before bedtime.

We focus on sensory issues, using sensory‐based activities to address sleep problems. (Participant 9)Participants used techniques such as deep compression therapy and massage to help children relax and prepare for sleep. These approaches were particularly useful for children who experienced sensory overload or difficulty settling down at night.

Sensory‐based therapy, such as deep compression and massage, is used to address sleep‐related issues. (Participant 8)A sensory diet that included activities like proprioception and vestibular exercises was another commonly used strategy. This type of intervention helped regulate the child’s sensory system and promoted better sleep.

We use a sensory diet, focusing on proprioception and vestibular activities, as part of the intervention for the sleep difficulties, such as deep compression, massage, or playground visits in the evening, to regulate the child’s energy levels before the transition to bed. (Participant 7)

##### 3.1.2.3. Code: Physical Activity.

Physical activity was incorporated to regulate the child’s energy levels during the day and improve sleep at night. Participants used gross and fine motor training to ensure that children were sufficiently engaged in physical activities throughout the day, which helped with sleep initiation and quality.

A lot of physical activity, including gross and fine motor training, is incorporated to ensure the child is physically engaged during the day, which helps them to sleep at night. (Participant 8)

I suggest physical activities in the evening to help prevent daytime naps. It is also important for them to be active during the day. (Participant 4)Additionally, a participant also combined reducing screen time and encouraging more active play, such as visiting the playground to ensure that the child engages in physical activity, which would help tire them out for the night.

We reduce their screen time and encourage activities like going to the playground. If they have a sibling, we encourage to involve them in play as well. These activities typically involve large movements, which help tire them out and prepare them for sleep. (Participant 4)

##### 3.1.2.4. Code: Environmental Strategies.

Environmental strategies were another key intervention to optimize sleep. These included introducing the 2‐h digital detox (limiting screens), followed by dimming household lights to signal the transition to sleep, room layout, and comfort levels within the child’s sleep environment. Participants educated parents on how to set up a sleep‐friendly environment, emphasizing the importance of dim lighting before sleep, as well as separating play areas from sleeping areas to reduce distractions.

We also pay attention to the lighting before bedtime, ensuring it is dim, and to the arrangement of the room. We also educate parents not to mix play areas with sleep areas. (Participant 1)

For example, before sleep, what does the child do? How do they sleep? Understanding these patterns allows us to suggest a suitable intervention. We educate the parents so they can provide the right environment or routine to support better sleep. (Participant 10)A participant also recommended comforting elements, such as decorative bedding or nightlights, to make the sleeping environment more appealing and conducive to sleep.

We suggest using calming, comfortable bedding and adding elements like nightlights to make the sleeping environment more appealing to the child. (Participant 3)

### 3.2. Theme 2: Perspective on the Intervention

The theme of perspective on the intervention explored OTs’ views on the effectiveness and significance of sleep interventions for children with ASD. Three subthemes were identified: belief in the intervention, benefits of the intervention, and value of the intervention.

#### 3.2.1. Subtheme 1: Belief in the Intervention

Participants expressed the belief that empowering parents to establish consistent routines and practices was essential for addressing sleep disturbances in children with ASD. They highlighted that when parents were provided with guidance and resources, they were better able to manage their child’s sleep‐related challenges.

##### 3.2.1.1. Code: Parent Empowerment and Consistency.

There was a shared belief among participants that consistent routines were crucial for children with ASD, particularly in helping them manage sleep. Several participants emphasized that empowering parents to establish a routine helped both the child and the parents.

When we assist the parents, they can manage the routine better on their own. (Participant 7)

In my opinion, when we teach them about the sleep diary and they implement it, it can help them to establish a consistent routine for their child. This is particularly helpful for children with autism, who tend to be more rigid. For instance, establishing a consistent routine, like turning off the lights at 10 PM, helps maintain structure. (Participant 8)Participants strongly believed that sensory regulation was another key intervention strategy. They discussed the positive effects of sensory input on regulating the child’s sleep–wake cycle and biological rhythms, thereby enhancing sleep quality. Sensory activities were considered essential in preparing children for sleep.

##### 3.2.1.2. Code: Sensory Regulation for Sleep.

Participants expressed the belief that providing sensory input could help regulate children’s biological processes, including the sleep–wake cycle, hormone production, and overall sleep quality. They stressed that sensory input, such as sensory diets or activities, played a vital role in improving sleep.

In my opinion, providing sensory input sends signals to the brain, helping regulate hormone imbalance and improving the sleep‐wake cycle. This also enhances the release of hormones. (Participant 6)

##### 3.2.1.3. Code: Belief in Improved Sleep Through Activity.

Participants emphasized the importance of physical activity during the day as a key factor in improving sleep quality at night. By making the child physically tired, participants believed that sleep would come more naturally and at an appropriate time.

We include physical activity during the day so that the child is physically tired and more likely to fall asleep earlier at night. (Participant 8)

When they engage in physical activities during the day, they will be tired at night, just like adults. When we are tired, we can sleep well. (Participant 4)

#### 3.2.2. Subtheme 2: Benefit of the Intervention

This subtheme captured the perceived positive effects of sleep interventions for children with ASD as described by the participants. Subthemes identified in the data included improved sleep quality and routine, increased functionality and academic performance, and reductions in parental stress and anxiety. The following sections describe the benefits associated with sleep interventions for children with ASD.

##### 3.2.2.1. Code: Improved Sleep Quality and Routine.

Participants emphasized that one of the primary benefits of sleep interventions was the improvement in sleep quality and the establishment of a consistent sleep routine. These interventions helped create a structure for children with ASD, enabling them to sleep better and more regularly. Participants also highlighted that sensory‐based interventions played a role in enhancing the quality of sleep by reducing sensory overload.

When sensory‐based interventions are applied consistently, the frequency of improvements increases. The more they do it, the more noticeable the progress. If it’s done less, the progress will be slower. (Participant 9)

When the child experiences good or deep sleep, it also improves the parent’s daily activities—emotionally and in terms of routine. When the child’s sleep improves, it positively impacts the family. (Participant 6)Participants believed that the improvement in sleep patterns not only benefited the children but also improved the overall family routine, highlighting the interconnectedness between the child’s sleep and the family’s daily functioning.

##### 3.2.2.2. Code: Increased Functionality and Academic Performance.

Another significant benefit identified was the improvement in the child’s functionality and academic performance. Participants believed that when children had consistent, quality sleep, they could perform better in both therapy and educational settings. Sleep was seen as a crucial factor in enhancing a child’s cognitive ability, including their attention span, memory, and behavior.

If the child gets enough sleep, their ability to perform during the therapy session improves. They can follow instructions and cooperate more effectively. (Participant 4)

The child will focus better at school, and as parents are concerned about academic performance. Good sleep patterns can improve behavior and academic outcomes. (Participant 8)Participants noted that sleep quality positively affected the child’s behavior, as well as their academic and social development, demonstrating the comprehensive impact of sleep on the child’s overall functioning.

##### 3.2.2.3. Code: Reduction in Parental Stress and Anxiety.

In addition to improvements in the child’s well‐being, reduced parental stress and anxiety were another key benefit. One participant noted that parents felt more in control and less anxious when their child’s sleep improved. They described how parents often expressed gratitude, feeling relieved from the emotional and physical strain caused by disrupted sleep.

When the child has good sleep quality, parents feel less stressed. They express gratitude, saying they are less worried about their child’s behavior. (Participant 4)

Parents have said that after improvements in sleep, they feel less pressured and can focus better on their own lives. They have expressed immense gratitude, saying, “Thank you, thank you.” (Participant 4)The participant observed that when children slept better, parents experienced less emotional strain, creating a more positive family dynamic and improving the overall quality of life for the family.

#### 3.2.3. Subtheme 3: Value of the Intervention

This subtheme focused on the perceived significance and effectiveness of sleep interventions for children with ASD. Participants shared strong beliefs about the value of routine management, sensory input, and structured approaches in improving sleep outcomes for children with ASD. The subthemes identified in the data included the importance of routine and consistency, guidelines and structured approaches, and targeted interventions for specific needs.

##### 3.2.3.1. Code: Importance of Routine and Consistency.

Participants emphasized the value of routines and consistency in managing sleep for children with ASD. A structured approach, especially related to sleep patterns and prebedtime activities, was believed to be key to improving sleep quality. Participants indicated that, when parents followed a consistent routine, children were more likely to establish better sleep habits, which ultimately led to improved overall functioning.

If parents do not follow the sleep routine, the child will struggle to maintain the proper bedtime and preparation before bed. (Participant 9)

I usually ask about the sleep routine, eating habits, and daily activities so that we can maximize the progress during intervention. (Participant 1)Participants believed that routine management was a foundational aspect of the intervention, highlighting that parent empowerment in applying and maintaining routines was essential for successful outcomes.

##### 3.2.3.2. Code: Guidelines and Structured Approaches.

A recurring theme was the importance of having structured guidelines and standardized approaches for sleep interventions. Participants highlighted that having clear, structured plans helped both the therapists and parents implement effective interventions at home, ensuring consistency in practice. This belief in structured guidelines was seen as crucial for achieving lasting improvements in sleep patterns.

Yes, if a guideline or module were specifying what needs to be done and for how long, I believe that would help OTs recommend it to parents for use at home. (Participant 3)

As other OTs mentioned earlier, managing sleep is part of our domain. We base it on our framework, and it is the responsibility of an OT to analyze sleep patterns in children. (Participant 5)Participants believed that clear, evidence‐based guidelines provided a framework to follow, making it easier to achieve the desired results and making interventions more accessible for parents to apply at home.

##### 3.2.3.3. Code: Targeted Interventions for Specific Needs.

Participants also highlighted the importance of targeting interventions to address specific sleep issues in children with ASD. These targeted interventions were designed to address the unique challenges each child faced, including reducing excessive daytime sleep or using visual cues for children who struggled with verbal instructions.

Typically, the first treatment I emphasize is sleep routine management. For example, if the child sleeps too much during the day, it can cause issues with sleeping at night. Therefore, we need to reduce daytime sleep. (Participant 2)

Many children with autism are visual learners, so they can have a better understanding of bedtime activities when they can see what they need to do. This helps us achieve our goals and helps the child understand their bedtime routine. (Participant 3)Participants emphasized that tailoring interventions based on the child’s specific needs, such as addressing sensory processing difficulties or behavioral issues, was essential for improving sleep. This personalized approach was believed to be more effective in achieving lasting improvements.

### 3.3. Theme 3: Collaborative Delivery of Sleep Intervention

The theme of referral to other professionals emerged organically from the discussions, despite not being a primary focus of the initial interview guide. Participants frequently underscored that their “perspectives” on sleep intervention naturally extended beyond the therapy room to include collaborative delivery through referral practices. This reflected a multidisciplinary approach that integrated medical, behavioral, and sensory treatments.

#### 3.3.1. Subtheme: Referral Practices

The participants identified a need for specialized treatment and medical diagnosis, particularly when sleep disturbances were linked to medical conditions or when behavioral and sensory interventions failed to address the root causes of the problems. In these cases, some participants commonly referred the cases to medical professionals to ensure comprehensive care.

##### 3.3.1.1. Code: Specialized Treatment and Medical Diagnosis.

One participant explained that they often referred parents to specialists when sleep issues required specific medical interventions:

Usually, it doesn’t lead to more serious problems unless there are cases that require treatment from other disciplines, such as medication. In those cases, I would advise parents to consult the relevant specialist. (Participant 2)In cases where participants observed that the child’s sleep disturbances might stem from underlying medical conditions such as hormonal imbalances or neurotransmitter issues, referrals to doctors for further medical diagnosis were recommended:

When they arrive here, we conduct an evaluation and may recommend referring them to a specialist for further consultation and diagnosis. (Participant 8)In one instance, a participant described a case where a child had difficulty sleeping due to a melatonin deficiency. Despite implementing sensory‐based therapies, the participant referred the child to a doctor for medical treatment:

Every child has unique characteristics, issues, and problems that need to be addressed. We must understand what calms them down. There must be something that helps calm them, but if sensory‐based therapy or rest doesn’t help, we may need to refer them to a doctor to check for neurotransmitter levels or hormone imbalances. For example, one of my students had a melatonin deficiency, and the doctor recommended supplements. The issue wasn’t related to sensory factors but occurred when he changed his routine after returning to his hometown. His rigid routine made it difficult for him to adjust back. Despite therapy and other interventions, the doctor suggested melatonin supplements. If he becomes too hyper, he gets overstimulated. (Participant 5)

## 4. Discussion

This qualitative study explored the sleep interventions used by pediatric OTs for children with ASD in Malaysia. The results provide valuable insights into the practices of OTs and suggest potential improvements in managing sleep disturbances for children with ASD. By comparing these findings with global literature, the study provides insights into how pediatric OTs in Malaysia perceive and implement sleep interventions in ways that align with, or diverge from, existing international practices. Additionally, the proposed interventions that emerged from the study offer important perspectives on enhancing the management of sleep disturbances in children with ASD, emphasizing the need for personalized, culturally appropriate, and holistic strategies.

### 4.1. Structure of the Interventions

Our participants emphasize the importance of frequency and intensity in sleep interventions, suggesting that OTs should tailor schedules based on the severity of sleep disturbances, time constraints, and parental involvement [20, 30]. Regular, consistent interventions—such as limiting screen time and adjusting the sleep environment—remain fundamental to promoting sleep hygiene. These findings align with established research highlighting the efficacy of structured routines and environmental modifications for children with ASD [31].

However, it is essential to distinguish the institutional culture of these participating private centers from the broader Malaysian national landscape. In the private sector, as observed in this study, OTs often possess the clinical “luxury of time” to provide extended parent education and frequent follow‐up interventions based on specific parental feedback. This contrasts with the high‐volume Malaysian public healthcare sector or rural CBRCs, where higher caseloads and limited resources may necessitate more streamlined or group‐based models.

The frequency of interventions in this study varied, with sessions offered daily or weekly, depending on the setting and availability. While more frequent interventions generally yielded better outcomes, follow‐up assessments remain vital for gauging long‐term efficacy [30]. Parental involvement was underscored as a critical determinant for maintaining consistency; OTs relied heavily on parental reporting to monitor progress and titrate adjustments [32, 33]. Consequently, while personalized intervention plans and scheduling flexibility are crucial for success, the perspectives identified here represent a snapshot of specialized private practice in northern Malaysia rather than a universal standard for the entire country. These findings are intended to offer transferability to similar private clinical contexts, providing a baseline for understanding how OTs in comparable settings navigate sleep disturbances in children with ASD.

### 4.2. Types of Sleep Interventions Implemented

The findings revealed that multimodal interventions were predominantly employed by some OTs in Malaysia. These included behavioral, sensory‐based, physical, and environmental strategies, each of which was used to address different aspects of sleep disturbances. Behavioral interventions, such as structured bedtime routines, visual schedules, and minimizing bedtime disruptions, were the most commonly implemented strategies. These interventions are consistent with global research, which shows that predictable routines improve sleep onset and quality for children with ASD [23, 31, 34]. In particular, a participant with over 7 years of experience emphasizes that the use of visual schedules has been identified as a particularly effective strategy, helping children with ASD follow a structured sequence of events, which can reduce anxiety and promote better sleep hygiene [23, 35, 36].

However, while behavioral strategies are widely recognized for their effectiveness, they often require significant consistency and family involvement to be successful [24]. Participants in this study revealed that they face challenges in ensuring these strategies are implemented consistently at home. This perceived “lack of commitment” likely stems from complex underlying barriers, such as socioeconomic constraints, limited knowledge of sleep hygiene, or the practical difficulty of maintaining Western‐centric routines within cosleeping households. The role of parents in implementing these behavioral interventions is crucial; however, commitment is frequently moderated by the feasibility and cultural relevance of the intervention itself. Further research is needed to assess how parent training and support can be improved to ensure long‐term success by addressing these specific contextual barriers [33, 34].

In addition to behavioral interventions, sensory‐based strategies, such as deep pressure therapy and proprioceptive exercises, were commonly used by the participants in this study. These interventions are aimed at regulating sensory input and helping children with ASD manage sensory issues, which is a common barrier to sleep [16, 21]. The use of sensory‐based intervention is supported by many in the literature, with studies indicating that sensory strategies can improve sleep outcomes by calming the nervous system and reducing sleep onset [30, 37–39]. Gringras et al. [40] and Lane et al. [41] reported that sensory‐based interventions are not always universally effective across all children with ASD, particularly in high‐quality studies. The sensory needs of children with ASD are highly individualized, which means that the interventions should be personalized to the child’s unique sensory sensitivities [42, 43]. The individualization of these strategies requires not only trained professionals but also the active involvement of parents who can adjust the strategies to suit their child’s specific needs. The emphasis on sensory‐based strategies over behavioral models like CBT‐I reflects a specific clinical reasoning among the participating OTs. Rather than viewing them as competing approaches, therapists prioritized sensory regulation as a physiological prerequisite for behavioral success. In this context, OTs perceive sensory dysregulation as a primary barrier that must be mitigated to stabilize a child’s arousal levels, thereby making them more receptive to structured behavioral routines like visual schedules or bedtime fading.

Environmental modifications, such as dimming lights, reducing noise, and creating a calming sleep space, were also commonly used by the participants [44]. These strategies are aligned with global best practices in improving sleep hygiene, where controlling the sleep environment is seen as one of the most effective ways to mitigate sleep disturbances [8, 45]. In this study, some participants emphasize that environmental factors such as lighting and noise can significantly impact a child’s ability to fall asleep and stay asleep [36, 46]. However, a critical point of concern is the cultural adaptation of such environmental modifications. A critical aspect of clinical utility in the Malaysian context is the tailoring of interventions to family dynamics. Participants noted that interventions must be “parent‐powered”; therapists act as educators who empower parents to manage routines independently. In practice, this means that OTs must negotiate recommendations—such as bedroom layout—with the cultural reality of cosleeping, which is prevalent in Malaysia [9]. Rather than mandating independent sleep, Malaysian OTs appear to focus on modifying the shared environment (e.g., bedding textures and lighting) to improve sleep quality within the existing family structure.

The use of physical activity as a sleep intervention was another strategy emphasized by participants in this study. They suggested incorporating both gross motor and fine motor activities throughout the day to help children expend energy, thus facilitating better sleep at night [20, 47]. This aligns with existing research, which shows that physical activity can help children with ASD achieve better sleep by regulating their energy levels and promoting relaxation [48, 49]. However, intensity and timing of physical activity need to be carefully considered, as excessive or poorly timed physical activity can sometimes result in increased arousal, making it harder for children to settle at night [50, 51]. Future research should explore the optimal type and timing of physical activity that best supports sleep for children with ASD.

### 4.3. Belief in the Intervention

A prominent theme that emerged was the belief in the effectiveness of consistent interventions, especially in empowering parents to establish and maintain structured sleep routines. Participants strongly believed that when parents were given the right guidance, such as sensory regulation strategies and consistent bedtime routines, they could significantly improve their child’s sleep. This aligns with existing literature that emphasizes the critical role of family involvement and the empowerment of parents in managing sleep disturbances in children with ASD [32, 52]. The participants’ belief in the sensory regulation approach also echoed findings from studies that have shown how sensory‐based interventions contribute to improved sleep by regulating the child’s biological sleep–wake cycle [38, 39]. However, a study suggested more cautious or even limited findings. For instance, a study by Gringras et al. [40] found that sensory interventions, specifically weighted blankets, did not produce significant improvements in sleep patterns among ASD children. While it may show positive results in some cases, there is not enough robust evidence to consider it universally effective for all children or sleep disturbances.

### 4.4. Benefits of the Intervention

The benefits of the interventions were evident in the improved sleep quality, which was consistently linked to reduced parental stress and better child behavior [22, 52]. As reported by the participants in this study, sleep interventions led to better sleep quality and regular sleep routines, which, in turn, had a positive impact on the child’s behavior, functionality, and even academic performance. These results resonate with studies that have highlighted the significant benefits of improved sleep for children with ASD, including reduced repetitive behavior and improved cognitive function [49, 53]. Additionally, the reduction in parental stress reported by participants is consistent with findings from other studies that suggest improved sleep leads to better family dynamics and reduced emotional burden on caregivers [23, 31, 32, 34]. It is also important to acknowledge that in Eastern cultures such as Malaysia, cosleeping is significantly more common than in Western contexts. Consequently, sleep interventions developed in the West—which often assume independent sleeping environments—may not be culturally congruent or effective without adaptation [9, 24]. Therapists must therefore tailor strategies that support family sleep goals while remaining respectful of local practices.

### 4.5. Value of the Intervention

Participants in this study strongly valued the importance of establishing consistent routines for children with ASD, noting that structure played a crucial role in helping these children manage their sleep. The value placed on routine and consistency in sleep interventions is supported by literature suggesting that children with ASD benefit greatly from predictable environments and schedules, which contribute to better sleep outcomes [54, 55]. Additionally, the participants emphasized the need for structured guidelines and targeted interventions tailored to the specific needs of the child, particularly in terms of sensory and behavioral strategies. This approach reflects findings from other studies highlighting the need for personalized care in managing ASD‐related sleep issues [18, 31, 56]. While the participants reported perceived positive outcomes, it is important to clarify that as a qualitative exploration, this study is aimed at informing clinical practice and serving as a conceptual foundation for future intervention development rather than proving clinical effectiveness. These findings highlight the specific strategies valued by Malaysian practitioners, providing a necessary contextual framework for subsequent quantitative trials to evaluate their impact on child and family outcomes.

### 4.6. Referral Practices

The emergence of referral practices as a theme provides a more comprehensive view of the occupational therapy perspective in Malaysia. It suggests that sleep intervention is not viewed by therapists in isolation, but as a component of a broader, integrated care model. This finding complements the primary aim of the study by illustrating that for complex ASD‐related sleep disturbances, the OT’s approach includes acting as a clinical gatekeeper to specialized medical care. While OTs provided foundational interventions, they frequently referred children to medical professionals when sleep issues were linked to underlying medical conditions, such as hormonal imbalances or required pharmacological approaches [20, 57]. This approach aligns with the multidisciplinary care models advocated by researchers [22] and emphasizes the importance of collaboration between OTs and other healthcare providers, such as pediatricians and sleep specialists, in managing complex sleep disturbances [8]. The referral practices described in this study reflect a holistic approach to care that considers the child’s medical, sensory, and behavioral needs, underscoring the importance of integrated care in improving sleep outcomes for children with ASD.

### 4.7. Clinical Implications and Framework: A Modular Approach to Sleep Management

The findings from this study provide a conceptual foundation for a modular sleep intervention framework specifically adapted for private pediatric practice in Malaysia. A core practice principle identified is the recognition of sleep as an occupation in cosleeping cultures; rather than mandating independent sleeping arrangements common in Western protocols, Malaysian OTs should focus on modifying the shared sleep environment, such as adjusting bedding textures and dimming household lights.

Furthermore, a structured OT decision pathway emerged, emphasizing sensory‐first readiness. In this pathway, therapists prioritize sensory regulation—using proprioceptive and vestibular “heavy work” like deep compression or evening playground visits—as a physiological prerequisite to stabilize a child’s arousal levels. Only once sensory regulation is achieved are behavioral parent education components introduced, such as the use of visual schedules and the “2‐h digital detox” to establish consistent bedtime routines. This modular approach, which also incorporates a clear referral trigger for medical consultation if strategies do not yield progress within 2 weeks, offers a testable clinical model for improving sleep participation in children with ASD within a culturally responsive context.

## 5. Limitations and Future Directions

This study provides valuable insights into the sleep interventions employed by pediatric OTs for children with ASD. However, several limitations must be considered. First, the study sample was recruited from two specific pediatric rehabilitation centers in northern Malaysia. While this limits the generalizability of the findings to all Malaysian OTs—particularly those in the public sector or rural hospitals—the study prioritizes transferability. By providing a rich, contextualized account of private practice in this region, the results offer valuable insights that may apply to therapists working in similar specialized pediatric settings. Future research should expand to include more diverse institutional cultures to further broaden these insights.

Another significant limitation is the reliance on self‐reported data from OTs. While focus groups capture valuable perspectives, they carry an inherent risk of social desirability bias, where participants may report perceived rather than actual clinical success. Furthermore, without data from parents or objective measures—such as standardized sleep diaries, actigraphy, or direct observations—the findings may reflect therapist recollections rather than real‐world outcomes in the home. Future research should incorporate these objective assessments and pre‐ and postintervention data to provide more robust evidence of efficacy. Specifically, to align with the OTPF‐4, future studies should utilize validated tools such as the Children’s Sleep Habits Questionnaire (CSHQ) to measure the domain of “Occupations” (sleep participation). Integrating these standardized measures will move the field from qualitative exploration toward objective, framework‐aligned clinical trials that evaluate how interventions impact “Performance Patterns” within the Malaysian healthcare system. Finally, while parental involvement was identified as a key factor, this study did not investigate the specific barriers parents face when implementing strategies at home, particularly in lower socioeconomic or rural contexts. Future studies should explore parent‐specific challenges and the accessibility of training programs to ensure the sustainable success of sleep interventions in the Malaysian community.

## 6. Conclusion

This qualitative study explored how pediatric OTs in Malaysia approach sleep interventions for children with ASD. The findings reveal a preference for multimodal strategies—combining behavioral, sensory‐based, and environmental approaches—supported by an emergent clinical perspective that emphasizes the necessity of collaborative, multidisciplinary referrals for complex cases. There is a strong emphasis on parental involvement and routine consistency, with results highlighting the contextual adaptation of global practices to Malaysia’s healthcare and cultural settings.

The results highlight the contextual adaptation of global practices within the private pediatric OT landscape of northern Malaysia. By illuminating these specific local practices, this study provides a foundational understanding that contributes to the broader knowledge of pediatric sleep interventions in non‐Western, specialized clinical settings. These insights provide a vital foundation for the development of culturally responsive modules, though further empirical research is required to establish the objective effectiveness of these strategies within the Malaysian healthcare system. Future research should therefore investigate how these interventions are applied across diverse regions and how therapist training impacts delivery.

## Author Contributions

All authors listed have made substantial contributions to the development, data collection, analysis, and writing of this study.

## Funding

No funding was received for this manuscript.

## Disclosure

This study is a small part of a PhD thesis entitled “The Development and Validation of Sleep Quality Enhancement Module for Autism Spectrum Disorder Children with Sleep Onset Delay: For Occupational Therapists.” All authors have approved the final version.

## Conflicts of Interest

The authors declare no conflicts of interest.

## Supporting information


**Supporting Information** Additional supporting information can be found online in the Supporting Information section.

## Data Availability

Data underpinning these findings are available from the corresponding author, A.D., upon reasonable request.
